# Cholesterol Granuloma of the Frontal Sinus: A Case Report

**DOI:** 10.1155/2012/515986

**Published:** 2012-10-24

**Authors:** Manola Marco, Casorelli Ida, Pietrafesa Francesco Luigi, Mottola Giampiero, Lacerenza Domenico, Battiloro Giuseppe, Patitucci Giuseppe, Vita Giulia Anna Carmen

**Affiliations:** ^1^Otorhinolaryngology Department, S. Giovanni di Dio Hospital, Via Foggia, 85025 Melfi, Italy; ^2^Ophthalmology Department, S. Francesco d'Assisi Hospital, S.S. Appia, 85029 Venosa, Italy; ^3^Anatomical Pathology Department, IRCCS-CROB, Via Padre Pio, 85028 Rionero in Vulture, Italy

## Abstract

Cholesterol granulomas are common in the mastoid antrum and air cells of the temporal bone. In the paranasal sinuses, especially in the frontal sinus, they have occasionally been mentioned in the literature. The pathogenesis is unknown, but the majority of the authors support the concept of airway obstruction in the cells well pneumatised of temporal bone and paranasal sinuses. The authors report a case of cholesterol granuloma of the frontal sinus treated with radical surgical techniques, and they also recommend an endoscopic approach to frontal sinus to restore or enlarge the nose-frontal canal and promote drainage and ventilation of the frontal sinus.

## 1. Introduction

The cholesterol granuloma is a histological entity consisting of granulation tissue in which large quantity of cholesterol crystals provoke foreign body giant cell formation [1–6]. The pathogenesis of cholesterol granuloma is unknown. Numerous ethiopathological hypothesis have been proposed, but the majority of the authors support the concept of airway obstruction in the cells well pneumatised of temporal bone and paranasal sinuses. Obstruction of the air cells leads to rupture of blood vessels and hemorrhage. Red blood cell degradation into cholesterol crystals produces a foreign body giant cell [1, 5–9].

Cholesterol granuloma is common in the mastoid antrum and air cells of the temporal bone [10]. It has also been reported in other parts of the skull, such as the maxillary sinuses and orbit [3, 5, 11, 12]. In the paranasal sinuses, especially in the frontal sinus, they have occasionally been mentioned in the literature [1, 2, 6, 13, 14].

We report a case of cholesterol granuloma of the frontal sinus. The tumor is treated with radical surgical techniques, but we also recommend an endoscopic approach to frontal sinus to restore or enlarge the nose-frontal canal and promote drainage and ventilation of the frontal sinus. 

## 2. Case Report 

An 82-year-old male was referred to our attention with a two-year history of gradually enlarging swelling of the left brow. On examination, the swelling appeared soft and floating, 3-4 cm large, painless, and was not associated with diplopia and any ocular symptoms. On additional detailed anamnesis there was history of rhinitis with nasal obstruction and nasal discharge; there was no story of trauma. Nasal endoscopy demonstrated bilateral enlarged inferior turbinates.

**Figure 1:**
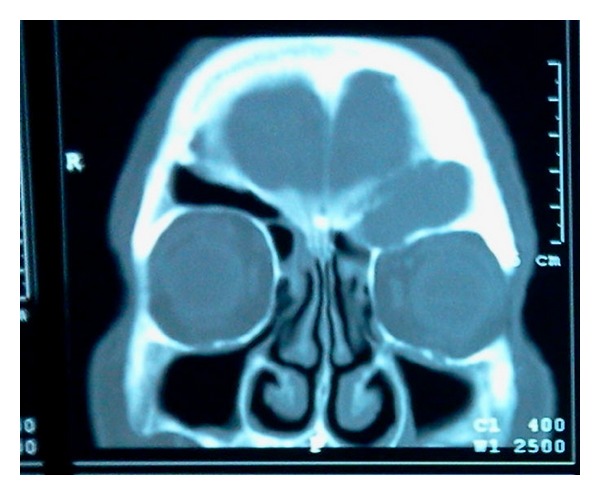
Computed tomography (CT scan) of the orbits shows complete opacity of the left frontal sinus with anterior and inferior breach of the sinus.

**Figure 2:**
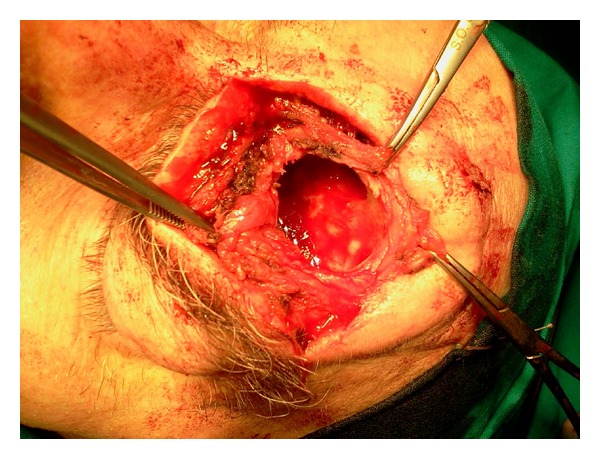
Intraoperative photograph shows the opening of the frontal sinus with Lynch-Howarth procedure.

**Figure 3:**
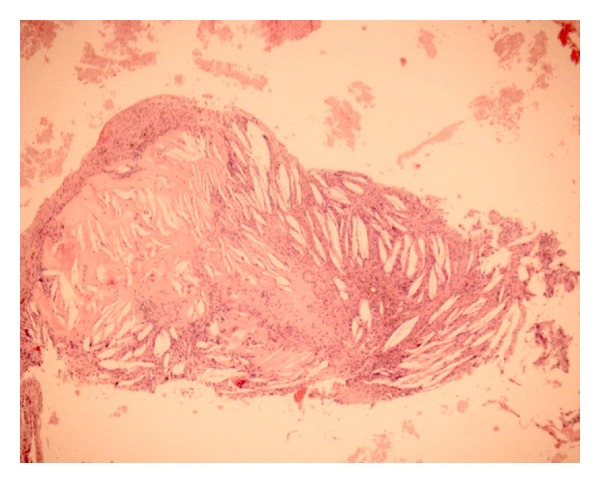
A histology slide of the cholesterol granuloma of the frontal sinus reported here. The fibrous connective tissue contains the characteristic “needles” of cholesterol surrounded by multinucleated foreign body giant cells (hematoxylin-eosin, original magnification ×50).

Computed Tomography (CT scan) of the orbits showed complete opacity of the left frontal sinus and partial opacification of contralateral frontal sinus and maxillary and ethmoidal sinuses. The opacification of the left frontal sinus appeared to be due to dense material without contrast enhancement which extended into the orbit through anterolateral (20 × 30 mm) and inferior (13 mm) breach of the sinus (Figure 1). Through Lynch-Howarth procedure, the frontal sinus was opened and the content was removed (Figure 2). In the second time of surgery, functional endoscopic sinus approach was performed to open the naso-frontal canal. Finally, plastic repair of the skull was done. The definite diagnosis is made by histology (Figure 3). We collected the informed consent of the patient to be published. 

## 3. Conclusions

Classically, the cholesterol granuloma is found in the petrous apex and other pneumatised areas of the temporal bone and paranasal sinuses. The pathogenesis is unknown, but many authors suggest that the key factors are prolonged inflammation and obstruction of a bony cavity that is normally aerated. Leon et al. suggested that the increased intrasinus pressure due to drainage obstruction may affect the venous and lymphatic drainage from the sinus cavity and cause the rupture of blood vessels and hemorrhage [5]. In this circumstance, the lymphatic drainage may be insufficient to completely remove the lipid components of the red blood cells, and the lipid accumulation may contribute to the formation of cholesterol crystals and their esters [2, 3, 5]. The formation of a cyst that slowly grows causes bone destruction and compression of the surrounding structures that lead to clinical symptoms. 

Cholesterol granuloma of the frontal sinus is uncommon. Our MIDLINE literature search on “cholesterol granuloma of the frontal sinus” has reflected the rarity of this condition. Butler and Grossenbacher [4] and Hellquist et al. [15] wrote that disordered ventilation and impaired drainage are decisive pathogenic factors in the causation of cholesterol granuloma. Shykhon et al. [9] and Ochiai et al. [16] supported the need for radical surgery to prevent recurrence.

**Figure 4:**
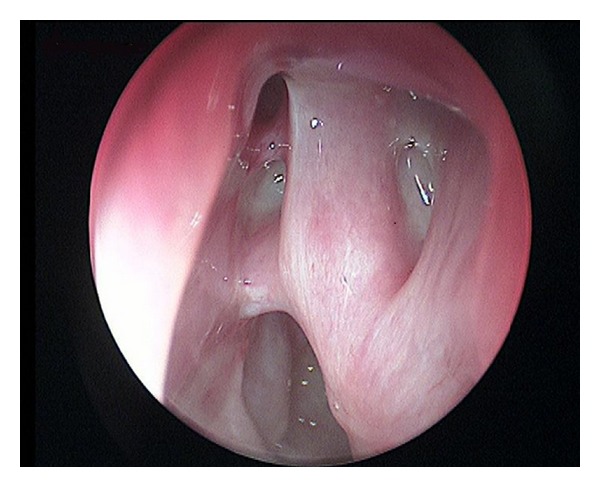
Postoperative results.

The case reported by us was treated with radical surgical techniques and with an endoscopic approach to frontal sinus to restore or enlarge the nose-frontal canal and promote drainage and ventilation of the frontal sinus. 12–18-month followup shows no clinical signs of recurrence (Figure 4).
